# Evaluating Carotid Plaque Stiffness with Ultrasound 2D Shear-Wave Elastography in Patients Undergoing Coronary Artery Bypass Grafting

**DOI:** 10.3390/diagnostics15030338

**Published:** 2025-01-31

**Authors:** Adel Alzahrani, Amjad Ali Alharbi, Amjad Khalid Alharbi, Asma Alkhaldi, Asseel Z. Filimban, Abrar Alfatni, Reham Kaifi, Ahmad Albngali, Mohammed Alkharaiji, Omar Alserihy, Salahaden R. Sultan

**Affiliations:** 1 Department of Diagnostic Radiology and Imaging, King Abdullah Medical City, Makkah 24246, Saudi Arabia; alzahrani.a7@kamc.med.sa (A.A.); 2 Department of Radiologic Sciences, Faculty of Applied Medical Sciences, King Abdulaziz University, Jeddah 21589, Saudi Arabia; 3 College of Applied Medical Sciences, King Saud bin Abdulaziz University for Health Sciences, Jeddah 22384, Saudi Arabia; 4 King Abdullah International Medical Research Center, Jeddah 22384, Saudi Arabia; 5 Medical Imaging Department, Ministry of the National Guard—Health Affairs, Jeddah 11426, Saudi Arabia; 6 Department of Public Health, College of Health Sciences, Saudi Electronic University, Riyadh 93499, Saudi Arabia; 7 Department of Radiology, King Abdulaziz University Hospital, King Abdulaziz University, Jeddah 21589, Saudi Arabia

**Keywords:** ultrasound, elastography, 2D shear wave, carotid plaque, echogenicity

## Abstract

**Background:** Coronary and carotid artery diseases are manifestations of a systemic atherosclerotic process, often coexisting in patients affected by both conditions. This association emphasizes the importance of evaluating both coronary and carotid atherosclerosis in high-risk individuals. Ultrasound 2D shear-wave elastography (2D-SWE) has shown promise as a noninvasive technique for assessing carotid plaque stiffness. This prospective pilot study aimed to assess carotid plaque stiffness in patients undergoing coronary artery bypass grafting (CABG) and those not scheduled for the procedure as a control group. **Methods:** 32 patients (17 CABG and 15 controls) were recruited, collectively presenting 43 carotid plaques. Bilateral carotid ultrasound was performed using a high-resolution linear transducer. Plaque stiffness was quantified via 2D-SWE, expressed in shear-wave velocity (SWV, m/s) and Young’s modulus (YM, kPa). Plaque characteristics, including GSM, were quantified. Intra-observer reproducibility was evaluated with intraclass correlation coefficients (ICCs) and Bland–Altman plots. Statistical differences and correlations were assessed using Mann–Whitney U and Spearman’s correlation tests. **Results:** Carotid plaques in the CABG group exhibited significantly lower stiffness compared to controls (median stiffness SWV: 3.64 m/s vs. 4.91 m/s, *p* < 0.0001; YM: 20.96 kPa vs. 72.54 kPa, *p* < 0.0001). ICCs demonstrated excellent reproducibility for stiffness measurements (SWV: ICC = 0.992; YM: ICC = 0.992), with minimal bias in measurements. A positive correlation was observed between 2D-SWE and GSM values (SWV: r = 0.343, *p* = 0.024; YM: r = 0.340, *p* = 0.026). **Conclusions:** Ultrasound 2D-SWE has shown promise as a reliable tool for quantifying carotid plaque stiffness, demonstrating high reproducibility and a significant correlation with GSM. The observed reduction in plaque stiffness among CABG patients highlights its potential as a valuable parameter for identifying high-risk plaques and assessing cerebrovascular risk in patients undergoing CABG.

## 1. Introduction

Cardiovascular diseases (CVDs) are a leading cause of mortality globally, with atherosclerosis—a chronic inflammatory condition characterized by lipid accumulation, fibrous tissue, and calcification in arterial walls—serving as the primary underlying cause [1,2,3]. This process begins with endothelial dysfunction, triggering inflammation, lipid oxidation, and immune responses, ultimately leading to plaque formation and instability [1,4,5]. Risk factors such as hypercholesterolemia, hypertension, obesity, and diabetes exacerbate these processes, with emerging contributors including disturbed sleep and environmental stress [6,7]. The global burden of CVDs is significantly growing. According to the Global Burden of Disease Study 2019, the prevalence of CVD cases nearly doubled from 271 million in 1990 to 523 million in 2019, with deaths increasing from 12.1 million to 18.6 million during the same period [8]. Ischemic heart disease and stroke are identified as the primary contributors to these alarming statistics. Similarly, the Global Burden of Disease Study 2017 reported over 17 million CVD-related deaths, with ischemic heart disease and stroke accounting for 85% of fatalities [9].

Coronary artery disease (CAD) and carotid artery disease (CD) are major contributors to cardiovascular events, sharing a common atherosclerotic origin characterized by plaque accumulation within arterial walls [10,11]. These plaques can obstruct tissue perfusion or become unstable, precipitating thrombotic or embolic events with potentially fatal consequences [12]. The systemic nature of atherosclerosis frequently results in a significant overlap between CAD and CD, as demonstrated by carotid intima-media thickness, which correlates linearly with CAD severity and serves as a reliable predictor of its presence when ≥1.0 mm [13]. Moreover, high-grade carotid stenosis has been strongly associated with a high prevalence of significant coronary artery stenosis [14]. This interplay underscores the importance of evaluating both conditions in high-risk individuals as their coexistence amplifies cardiovascular risk and poses clinical challenges. Management strategies for coexisting CAD and CD must be carefully tailored to patient risk profiles. Staged procedures, such as carotid reconstruction followed by coronary artery bypass grafting (CABG), are often preferred for stable cases as they allow for the separate management of each condition, minimizing perioperative complications. Combined surgeries, such as carotid endarterectomy (CEA) with CABG, may be necessary for patients with active neurological symptoms or bilateral carotid lesions despite the increased risk of perioperative complications [15,16,17,18]. Minimally invasive options, including coronary angioplasty and carotid stenting, have shown promising results in appropriately selected patients, with low rates of neurological events, myocardial infarction, and mortality [19]. Comprehensive assessment and individualized care are essential to optimizing outcomes in these patients. The prevalence of coexistent CAD and CS ranges from 2% to 14%, with approximately 8% of patients undergoing CABG exhibiting significant stenosis in the extracranial carotid arteries [20]. This overlap is clinically significant as patients with both conditions are at a heightened risk of cerebrovascular complications. Consequently, an integrated approach to managing CAD and CS, which considers the systemic nature of atherosclerosis and incorporates advanced diagnostic and therapeutic modalities, is crucial for improving outcomes in this high-risk population. Advanced imaging and risk stratification tools facilitate better treatment planning and enable the evaluation of carotid plaque characteristics alongside the degree of stenosis, which may enhance risk assessment and stratification [21].

Ultrasound is a non-invasive, real-time, and cost-effective imaging tool used for evaluating carotid artery stenosis and plaque features, providing information about plaque morphology and composition, which are crucial for accurate cardiovascular risk assessment and effective clinical management [22]. Histopathological examinations showed that unstable carotid plaques are characterized by a thin fibrous cap, a lipid-rich necrotic core, intraplaque hemorrhage, and intraplaque neovascularization [23,24]. B-mode ultrasound imaging and Doppler ultrasound imaging are highly effective tools for evaluating carotid plaques. B-mode ultrasound provides detailed imaging of plaque morphology, allowing for the evaluation of echogenicity using the grayscale median (GSM) in which echogenic plaques are often fibrous and calcified, while echolucent plaques, which contain lipids or intraplaque hemorrhage, are more prone to rupture and associated with higher risk [25,26], suggesting that GSM holds significant potential as a valuable clinical tool in assessing plaque vulnerability and predicting future events. Doppler ultrasound imaging, including color and spectral Doppler modes, provides a hemodynamic assessment of blood flow parameters with the ability of quantifying the degree of carotid stenosis [27]. Ultrasound 2D shear-wave elastography (2D-SWE) is a promising ultrasound imaging technique that quantifies tissue elasticity [28,29]. Using acoustic radiation force impulse (ARFI) technology, 2D-SWE generates shear waves that propagate through tissue, with their velocity proportional to tissue stiffness [30,31]. Tissue stiffness can be quantified using Young’s modulus (YM = 3ρc^2^, where ρ is tissue density, and c is shear-wave velocity), and measured in YM (kPa) or velocity (m/s) [28]. This method has proven valuable in evaluating pathologies across various organs [32], including the assessment of liver fibrosis [33] and distinguishing between benign and malignant lesions in breast and thyroid nodules [34,35], enhancing diagnostic accuracy when combined with traditional ultrasound methods. Ultrasound SWE enables a real-time, colorimetric map of plaque stiffness, offering quantitative data that may facilitate risk stratification in patients with carotid artery disease [36]. A number of studies have confirmed the feasibility of ultrasound SWE in assessing carotid plaque and its correlation with clinical factors, demonstrating that lower YM values in vulnerable plaques compared to stable ones, as corroborated by studies examining histological markers of vulnerability, including fibrous cap thickness and lipid content [37,38]. Although ultrasound SWE has demonstrated potential in assessing carotid plaque, its specific application in patients undergoing CABG is yet to be investigated. Therefore, this study aimed to assess carotid plaque stiffness using ultrasound 2D-SWE in patients undergoing CABG compared to those without coronary heart disease symptoms and not scheduled for the procedure as a control group. Additionally, the study assessed the intra-observer reproducibility of ultrasound 2D-SWE in measuring carotid plaque stiffness and its correlation with GSM.

## 2. Methods

### 2.1. Study Design and Participants

This observational pilot study was ethically approved by the Institute Review Board of Research Ethics Committee at King Abdullah Medical City (IRB No. 23-1177). The study design adhered to the principles outlined in the Declaration of Helsinki and the International Conference of Harmonisation of Good Clinical Practice. Adult patients referred to the ultrasound department for carotid examination including those scheduled for CABG were recruited, and written informed consent was obtained. Patients with no carotid plaque detected via ultrasound, small plaque (i.e., with a circumference <3 mm), plaque obscured with acoustic shadowing, or those unable to give informed consent were excluded from the study. Patient demographic and clinical information, including age, gender, BMI, history of smoking, hypertension, and diabetes were collected. Ultrasound examination of carotid artery was performed in two visits with 1–2 weeks prior to surgery by an experience vascular scientist with efficient training in ultrasound 2D-SWE.

### 2.2. Ultrasound Assessment and Data Acquisition

**Figure 1:**
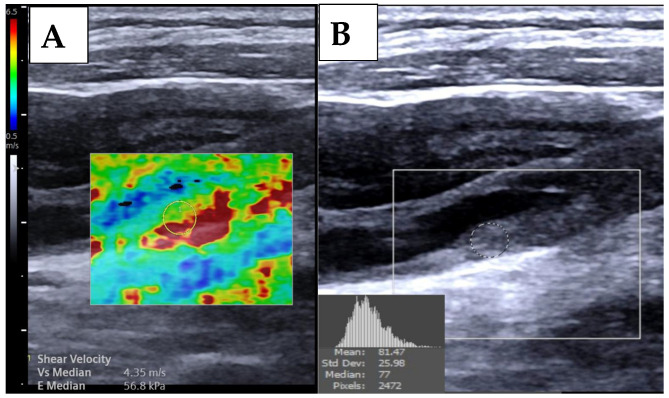
Measurements of ultrasound 2D-SWE (**A**) and grayscale median (**B**) from the carotid plaque.

Bilateral carotid ultrasound examinations were performed on all participants to evaluate the common, internal, and external carotid arteries using a high-resolution ACUSON Redwood ultrasound imaging system (Siemens Healthineers, Erlangen, Germany) equipped with a 10–4 MHz linear transducer. Image quality was optimized by adjusting key parameters, including depth, focus point, overall gain, time-gain compensation, and dynamic range, to achieve high-resolution B-mode images. Longitudinal views of all plaques were acquired for comprehensive assessment. Ultrasound 2D-SWE was used to measure carotid plaque stiffness, expressed in shear-wave velocity (m/s) and YM (kilopascals, kPa), for all plaques depicted via ultrasound with a circumference >3 mm protruding into the carotid artery lumen. Two dimensional SWE imaging settings were standardized according to established guidelines [39]. During ultrasound 2D-SWE imaging, participants were positioned supine with their neck slightly extended and arms resting comfortably at their sides. A sufficient amount of gel was applied, and the transducer was placed on the carotid artery with minimal pressure to ensure optimal contact and avoid compression artifacts. Dual-screen mode was activated, displaying the B-mode image alongside the overlaid elastography map, to facilitate precise placement of the region of interest (ROI) at the region of maximum plaque thickness to ensure reliable data acquisition. Plaque stiffness measurements were conducted twice, with a week interval between measurements for intra-observer reproducibility and agreement evaluation. For each session, five stiffness measurements were obtained per plaque, and the average of ten measurements across the two sessions was used to calculate plaque stiffness (total *n* = 320). For patients scheduled for CABG, these assessments were performed two weeks prior to surgery. The circular ROI for the SWE measurement was carefully positioned at the thickest part of the plaque, ensuring that the measurement reflected the stiffness of the plaque itself and excluded contributions from surrounding tissue or blood (Figure 1A). B-mode images in the longitudinal view were analyzed off-line using ImageJ 1.53 software to measure plaque length, thickness, and area. The DoS was determined based on the European Carotid Surgery Trial criteria [40]. GSM using Adobe Photoshop CC 2017 software [41] was determined by placing the ROI on the same location where the 2D-SWE measurements were taken (Figure 1B). Each B-mode ultrasound image was normalized using automatic linear scaling in Adobe Photoshop software in which the gray scale was calibrated such that a value of 0 represented the blood region, while higher values, indicating a whiter pixel intensity, were assigned to the adventitia region on a 255-point scale [42,43]. The average of ten measurements was used to calculate plaque GSM (total *n* = 320). Measurement of ultrasound 2D-SWE of plaque stiffness and GSM were performed by two different observers and were kept blinded to patient clinical information and their own measurements and those of the other observer to ensure unbiased data collection.

### 2.3. Statistical Analysis

Statistical analysis was conducted using SPSS Statistics (Version 21.0, IBM Corp., Armonk, NY, USA) and GraphPad PRISM 7 (GraphPad Software, La Jolla, CA, USA) for figure generation. Given the small sample size and the non-normal distribution of the data determined through histograms and the Shapiro–Wilk test, which revealed deviations from normality, non-parametric tests were selected. Mann–Whitney U test was used to determine whether there was a significant difference between DoS, size, and 2D-SWE of carotid plaques in CABG patients and non-CABG control groups. Intra-observer reproducibility and limits of agreement (LoAs) of plaque stiffness were evaluated using the intraclass correlation coefficient (ICC) and Bland–Altman plots. The correlation between plaque stiffness and GSM was assessed using Spearman correlation coefficient. Statistical significance was defined as a *p*-value ≤ 0.05.

## 3. Results

### 3.1. Patient Clinical Details and Carotid Plaque Characteristics

A total of 43 carotid plaques from 32 patients (17 CABG and 15 control) with a median age of 67 years were assessed via ultrasound. Of the 32 patients included in this study, 21 were male (12 CABG and 9 control) and 11 were female (5 CABG and 6 control), 25 had hypertension (14 CABG and 11 control), 24 were diabetic (12 CABG and 12 control), and 11 were smokers (4 CABG and 7 control). The median carotid plaque stiffness results were 3.85 m/s (3.64 CABG and 4.91 control) and 44.53 kPa (20.96 CABG and 72.54 control). The median DoS and plaque size were as followed: DoS 37.97% (34.78 CABG and 39.28 control), plaque length 1.12 cm (1.17 CABG and 0.97 control), plaque thickness 0.31 cm (0.31 CABG and 0.32 control), area 0.27 cm^2^ (0.30 CABG and 0.25 control), respectively. There were no significant differences in DoS (Z = −1.15, *p* = 0.24) nor plaque size (plaque length Z = −1.36, *p* = 0.17; plaque thickness Z = −0.02, *p* = 0.98; plaque area Z = −1.10, *p* = 0.26) between CABG and non-CABG groups. Patient clinical details and carotid plaque characteristics are summarized in Table 1.

### 3.2. D-SWE of Carotid Plaques

**Figure 2:**
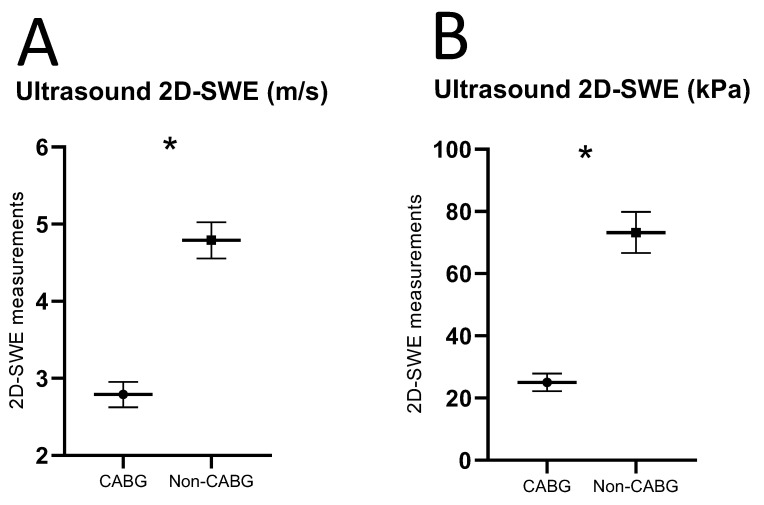
Ultrasound 2D-SWE of carotid plaques in m/s (**A**) and kPa (**B**) (mean ± SEM). * *p* ≤ 0.05 using Mann–Whitney U between carotid plaques from CABG and non-CABG control patient groups.

**Figure 3:**
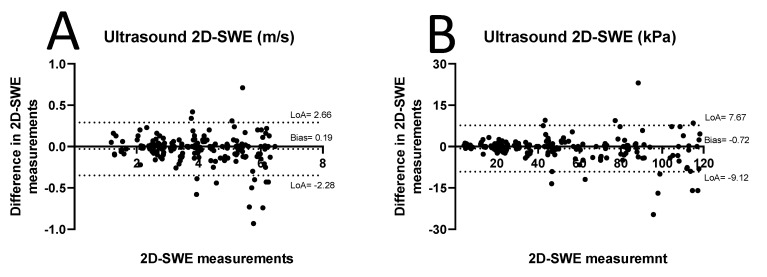
Bland–Altman inter-observer agreement of carotid plaque 2D-SWE in m/s (**A**) and kPa (**B**) measurements. LOA = limit of agreement.

Ultrasound 2D-SWEs of carotid plaques in the CABG group were significantly lower compared with the non-CABG control group (2D-SWE in SWV: CABG median 3.64 m/s, interquartile range (IQR) 1.02 m/s; control median: 4.91 m/s, IQR 1.69 m/s, Z = −4.76, *p* < 0.0001, Figure 2A; 2D-SWE in YM: CABG median 20.96 kPa, IQR 17.30 kPa; control median: 72.54 kPa, IQR 48.39, Z = −4.76, *p* < 0.0001, Figure 2B). The inter-observer agreement of ultrasound 2D-SWE in SWV and YM values was excellent, with ICC values of 0.992 (95% CI 0.990–0.994, *p* < 0.0001) and 0.992 (95% CI 0.990–0.994, *p* < 0.0001). The narrow confidence intervals (95% CI: 0.990–0.994) demonstrate a high degree of precision and reliability in the measurements and consistent across repeated measurements with minimal variability. The bias in 2D-SWE measurements in SWV was −0.03 ± 0.16 m/s (limits of agreement [LoA]: −0.35–0.29, Figure 3A). Bias in 2D-SWE measurements in YM was −0.72 ± 4.28 kPa (LoA: −9.12–7.67, Figure 3B).

### 3.3. Correlation Between 2D-SWE and GSM of Carotid Plaques

**Figure 4:**
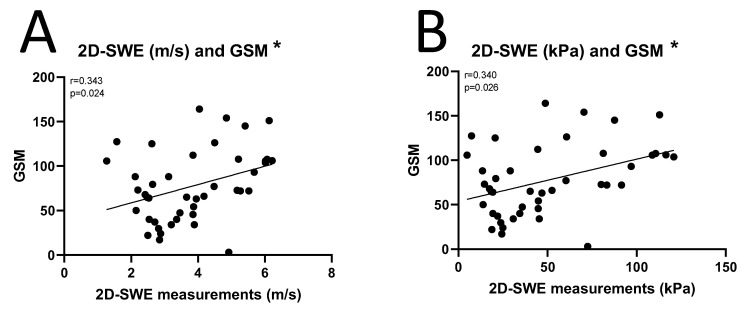
Correlation between 2D-SWE in m/s (**A**) and kPa (**B**) and grayscale median (GSM) of carotid plaques. * *p* ≤ 0.05 using Spearman’s rank correlation.

There was a significant positive correlation between 2D-SWE and GSM of carotid plaques (2D-SWE in SWV and GSM: Spearman’s rho coefficient 0.343, *p* = 0.024, Figure 4A; 2D-SWE in YM and GSMs: Spearman’s rho coefficient 0.340, *p* = 0.026, Figure 4B).

## 4. Discussion

Coronary and carotid artery diseases are common manifestations of systemic atherosclerosis and frequently coexist in high-risk individuals, underscoring the importance of thoroughly evaluating the carotid artery in patients undergoing CABG. This approach enhances cerebrovascular risk stratification and facilitates personalized management strategies, particularly for patients with coexistent coronary and carotid artery disease, where tailored interventions are essential to optimizing outcomes and minimizing perioperative complications. In addition to the ability of ultrasound to assess carotid stenosis severity and plaque characteristics [22,23,24,25,26,27,28,29,30,31,32,33,34,35,36,37,38,39,40,41,42,43,44], 2D-SWE offers a direct evaluation of tissue stiffness, thus it may enhance the stratification of cerebrovascular symptoms and events in patient with carotid plaque. To our knowledge, this is the first study that has evaluated carotid plaque stiffness using ultrasound 2D-SWE in patients undergoing CABG compared to a control group. The findings demonstrated significantly lower carotid plaque stiffness in CABG patients, suggesting potential differences in carotid plaque composition between groups. The study revealed a significant positive correlation between stiffness and GSM quantitative values, supporting the hypothesis that plaque stiffness correlates with echogenic characteristics. Additionally, the study showed the high intra-observer reproducibility of 2D-SWE measurements. These results underscore the promise of 2D-SWE as a non-invasive, accessible tool for stratifying cerebrovascular risk in high-risk populations, particularly those with coexistent coronary and carotid artery disease. By enabling a more precise characterization of plaque morphology and vulnerability, 2D-SWE may contribute to improved diagnostic accuracy and tailored management strategies, including the selection of appropriate surgical or medical interventions to mitigate the risk of stroke and other complications. Future research is needed to validate these findings in larger, multicenter cohorts and to explore the clinical implications of 2D-SWE in decision-making, including its role in guiding the treatment strategies and long-term monitoring of patients with systemic atherosclerosis. This would help to establish standardized protocols and maximize the clinical utility of this promising technology.

Our findings in CABG patients revealed a mean YM of 25.05 ± 13.04 kPa, significantly lower than the control group’s YM of 73.23 ± 31.04 kPa. These results align with prior research associating lower plaque stiffness with markers of unstable symptomatic carotid plaques. For instance, vulnerable plaques have been reported with YM values of 50.0 ± 19.6 kPa compared to 79.1 ± 33.8 kPa in stable plaques [38], while symptomatic plaques demonstrated YM values of 81.13 ± 20.12 kPa versus 115.78 ± 26.66 kPa for asymptomatic plaques [45]. Similarly, unstable plaques showed YM values of 50.0 ± 19.6 kPa compared to 79.1 ± 33.8 kPa in stable plaques [37], and symptomatic plaques had YM values of 62 ± 6 kPa versus 88 ± 9 kPa in asymptomatic plaques [46]. However, YM values appear to vary across studies, likely due to differences in SWE devices and technical factors such as acquisition protocols, imaging settings, and the type of velocity analysis employed. Procedural variations, including imaging plane selection and the impact of pulsatile arterial movements caused by blood flow, may also contribute to discrepancies [30,31,47,48,49,50]. This highlights the need for standardized SWE acquisition protocols considering these factors [51]. Based on the reported findings, the range of YM values for unstable plaques is approximately 25.05–62 kPa, while stable plaques generally exhibit YM values in the range of 73–115.78 kPa. This proposed threshold should be considered alongside the stenosis degree and plaque characteristics that are already widely incorporated in stroke risk assessment [22,23,24,25,26,27,28,29,30,31,32,33,34,35,36,37,38,39,40,41,42,43,44].

Despite this, SWE faces unique challenges in the context of carotid plaques due to their small size, morphological heterogeneity, and the dynamic pulsatile environment of the arterial wall [46,47,48]. Our analysis showed excellent reproducibility. Reproducibility has been corroborated by other studies, which report good to excellent agreement for carotid plaque [45,46]. In vitro studies using vessel stenosis phantoms have further validated SWE’s reliability, demonstrating its ability to differentiate soft and hard plaque regions with high accuracy and reproducibility [50]. Furthermore, SWE has demonstrated strong concordance with other imaging techniques, such as computed tomography angiography and pulse wave velocity, [38,39,40,41,42,43,44,45,46,47,48,49,50,51,52], and significant correlations with histopathological findings [37,38,39,40,41,42,43,44,45].

GSM analysis of carotid plaques on ultrasound provides valuable insights into plaque composition and correlates with the tissue composition determined histopathologically [53,54]. It has been shown that symptomatic patients had significantly lower GSM than asymptomatic patients, supporting its potential to identify vulnerable plaques [41]. Previous research reported a strong correlation between YM and GSM, further linking plaque echogenicity to its biomechanical properties where low GSM (hypoechoic) plaques, indicative of lipid-rich vulnerable plaques, consistently demonstrate lower SWV and YM values, while high GSM (hyperechoic) plaques, indicative of calcified stable plaques, exhibit higher stiffness [55,56]. The present study analysis also showed a significant positive correlation between the 2D-SWE and GSM of carotid plaques. Together, these findings highlight the potential of carotid plaque stiffness, assessed through ultrasound 2D-SWE, as a useful parameter for identifying high-risk plaques and assessing cerebrovascular risk in patients undergoing CABG.

Biomechanical factors are pivotal in the development, progression, and rupture of atherosclerotic plaques. The composition and morphology of plaques significantly affect their biomechanical characteristics and susceptibility to rupture [57,58]. This study corroborates these findings, demonstrating a correlation between plaque GSM and 2D-SWE-derived stiffness metrics. Plaques with a higher lipid content and larger necrotic cores typically exhibit lower stiffness compared to calcified or fibrous plaques, indicating a more vulnerable phenotype associated with cardiovascular events [59]. Established evidence highlights the association between carotid atherosclerosis and the extent and severity of coronary artery disease (CAD) [60,61]. Inflammation within atherosclerotic plaques contributes to systemic effects, with active inflammation in one vascular bed potentially exacerbating inflammation elsewhere. The necrotic core, characterized by the loss of extracellular matrix and replacement with lipid-rich debris and dead cells, represents the most vulnerable region within plaques, often linked to ongoing inflammation [62,63]. This vulnerability was further highlighted in a study by Sondore et al. (2023), which identified significant correlations between carotid and coronary plaque compositions using virtual histology-intravascular ultrasound [64]. Their findings emphasized that individuals with vulnerable coronary plaques are more likely to exhibit similar patterns in carotid plaques, underscoring the interconnected nature of systemic atherosclerosis [64].

The treatment of carotid plaques in patients undergoing CABG is a complex process requiring careful consideration of the interplay between carotid and coronary artery disease [65,66]. Treatment strategies must balance the need to reduce carotid plaque vulnerability and stenosis while minimizing risks, with the choice of treatment depending on factors such as the degree of carotid stenosis, plaque characteristics, the presence of neurological symptoms, and the urgency of CABG [12,13,14,15,16,17,18,19,20,21,22,23,24,25,26,27,28,29,30,31,32,33,34,35,36,37,38,39,40,41,42,43,44,45,46,47,48,49,50,51,52,53,54,55,56,57,58,59,60,61,62,63,64,65,66]. Staged and combined procedures are commonly employed in managing carotid plaques in CABG patients [67]. In staged procedures, carotid revascularization is performed first, either through carotid endarterectomy (CEA) or carotid artery stenting (CAS), followed by CABG at a later date. This approach reduces the risk of stroke by addressing significant carotid stenosis or unstable plaques before the systemic stress of CABG [66,67,68]. CAS is particularly advantageous in high-risk surgical candidates due to its minimally invasive nature and shorter recovery time compared to CEA [66]. Combined procedures, such as concurrent CEA and CABG, are typically reserved for patients with severe bilateral carotid stenosis, symptomatic carotid disease, or unstable plaques, despite the higher risk of perioperative complications [69].

This study has several limitations to consider. Patients with small plaques were excluded due to the fixed minimum ROI size (i.e., 3 mm) in the ultrasound imaging system used in this study. The ROI was placed on the thickest area of the plaque for consistency. This could have influenced the SWE and GSM values obtained for carotid plaques. However, standardized ROIs for measurements are essential to enhance the reproducibility. Additionally, plaques obscured due to acoustic shadowing were excluded because 2D-SWE relies heavily on the quality of underlying B-mode images, and limitations in these images directly affect SWE measurements. Furthermore, although the sample size of this study is relatively small, the included cohort effectively represents the typical clinical and demographic profiles of CABG patients. This representation encompasses prevalent comorbidities such as hypertension and diabetes, as well as age ranges commonly observed in this population. This alignment reinforces the relevance and applicability of the study’s findings to the broader CABG patient demographic. However, future studies with larger sample sizes are warranted to validate these findings and further enhance their generalizability.

## 5. Conclusions

This study underscores the utility of ultrasound 2D-SWE as a reliable and reproducible imaging modality for assessing carotid plaque stiffness in patients undergoing CABG, offering valuable insights into carotid plaque characteristics in individuals with coexisting coronary and carotid artery disease. Our findings revealed significantly reduced carotid plaque stiffness in the CABG group compared to controls, suggesting notable compositional differences and indicating that carotid plaques in CABG patients may be more vulnerable. Furthermore, the positive correlation between plaque stiffness and GSM quantitative values highlights the interplay between the biomechanical properties and composition of carotid plaques. These results emphasize the importance of combining quantitative stiffness measurements with echogenicity analysis to improve the characterization of plaque vulnerability, providing a more comprehensive understanding of the relationship between plaque mechanics and composition. These findings reinforce the potential of 2D-SWE to enhance cerebrovascular risk stratification by offering detailed assessments of carotid plaque vulnerability in high-risk patients with coexisting coronary and carotid artery disease. Future research involving larger, multicenter cohorts is crucial to validate these findings, explore their clinical implications, and establish 2D-SWE as a valuable tool for guiding decision-making and enabling personalized management strategies for this vulnerable population.

## Figures and Tables

**Table 1:** Patient clinical details and carotid plaque characteristics.

	Total	CABG	Control	*p*-Value
Patient, *n* [%]	32 [100]	17 [53.13]	15 [46.88]	-
Male, *n* [%]	21 [65.63]	12 [70.59]	9 [60]	-
Age, median [IQR, years]	67 [9.25]	68 [12]	67 [13]	0.77
Height, median [IQR, cm]	160 [8.75]	161 [10]	160 [11]	0.79
Weight, median [IQR, kg]	71 [12.25]	70 [14]	71 [9]	0.82
BMI, median [IQR, kg/m^2^]	27.05 [7.31]	26.95 [4.43]	30 [7.85]	0.79
Hypertension, *n* [%]	25 [78.13]	14 [82.35]	11 [73.33]	-
Diabetes, *n* [%]	24 [75.00]	12 [70.59]	12 [80.00]	-
Smoking, *n* [%]	11 [34.38]	4 [23.53]	7 [46.67]	-
Plaque, *n* [%]	43 [100]	21 [48.83]	22 [51.16]	-
DoS, median [IQR, %]	37.97 [19.12]	34.78 [18.10]	39.28 [20.94]	0.24
Plaque length, median [IQR, cm]	1.12 [0.67]	1.17 [0.56]	0.97 [0.70]	0.17
Plaque thickness, median [IQR, cm]	0.31 [0.11]	0.31 [0.11]	0.32 [0.11]	0.98
Plaque area, median [IQR, cm]	0.27 [0.25]	0.30 [0.24]	0.25 [0.26]	0.26
2D-SWE, median [IQR, m/s]	3.85 [2.54]	3.64 [1.02]	4.91 [1.69]	<0.001
2D-SWE, median [IQR, kPa]	44.53 [59.48]	20.96 [17.30]	72.54 [48.39]	<0.001

Abbreviation: BMI: body mass index, DoS: degree of stenosis, IQR: interquartile range, *n*: number, SD: standard deviation, SWE: shear-wave elastography.

## Data Availability

The data presented in this study are available on request from the corresponding author due to ethical reasons.
